# Ectoparasites of murids in peninsular Malaysia and their associated diseases

**DOI:** 10.1186/s13071-015-0850-1

**Published:** 2015-04-29

**Authors:** Siti Nursheena Mohd Zain, Syed Arnez Syed Khalil Amdan, Kamil A Braima, Noraishah M Abdul-Aziz, John-James Wilson, Paramesvaran Sithambaran, John Jeffery

**Affiliations:** Institute of Biological Sciences, Faculty of Sciences, University Malaya, 50603 Kuala Lumpur, Malaysia; Yayasan Peneraju Pendidikan Bumiputera, 15-1, Mercu UEM, Jalan Stesen Sentral 5, Kuala Lumpur Sentral, 50470 Kuala Lumpur, Malaysia; Department of Parasitology, Faculty of Medicine, University of Malaya, 50603 Kuala Lumpur, Malaysia; Museum of Zoology, Institute of Biological Sciences, Faculty of Sciences, University Malaya, 50603 Kuala Lumpur, Malaysia; Institute for Medical Research, Jalan Pahang, 50588 Kuala Lumpur, Malaysia

**Keywords:** Ectoparasites, Host distribution, Mites, Ticks, Fleas, Lice

## Abstract

A considerable number of rat-borne ectoparasite studies have been conducted since the early 1930s in the Malayan Peninsula (now known as peninsular Malaysia). The majority of studies were field surveys and collections of specimens across the region, and were conducted primarily to catalogue the ectoparasite host distribution and discover novel species. This has generated a signification amount of information, particularly on the diversity and host distribution; other aspects such as morphology, host distribution and medical significance have also been investigated. Amongst the four main groups (mites, fleas, ticks, lice), rat-borne mites have received the most attention with a particular emphasis on chiggers, due to their medical importance. More recent studies have examined the distribution of ectoparasites in rats from different habitat type simplicating a high prevalence of zoonotic species infesting rat populations. Despite being capable of transmitting dangerous pathogens to human, the health risks of rat-borne ectoparasites appear to be small with no serious outbreaks of diseases recorded. Although an extensive number of works have been published, there remain gaps in knowledge that need to be addressed, such as, the distribution of under studied ectoparasite groups (listrophorids and myobiids), determining factors influencing infestation, and understanding changes to the population distribution over time.

## Background

Rats have successfully exploited a wide variety of habitats and environments throughout the world. In many places, commensal rodents have adapted to living in close association with humans, using human agriculture products and waste as their food resources and buildings as their homes. “You are never more than 6 ft away from a rat” may be an urban myth, but demonstrates the intimate relationship between rats and human urban dwellers [1]. Rats, particularly species that live close to humans, play an important role in public health and the economy. Zoonotic diseases transmitted directly by rats through bites, urine and faeces include Leptospirosis [2], which affects 7-10 million people per year globally. However, perhaps more important is the indirect transmission of diseases that takes place through rat-borne ectoparasites such as the flea, *Xenopsylla cheopis*; the vector of causative agents, *Yersinia pestis*, responsible for the plague, and *Rickettsia typhi*, responsible for typhus [3].

Historically, collections and surveys of ectoparasites across the region now known as peninsular Malaysia, have been conducted to record and catalogue the various local rat-borne ectoparasite species [4-8], to determine their geographical distribution and host specificity [9-11], and in particular collect data regarding the incidence of medically significant species [12,13]. Since the 1940s, a significant amount of data has been accumulated on diversity and host distributions of rat-borne ectoparasites but surveys have, for pragmatic reasons, mostly focused on restricted taxonomics groups (e.g. ticks [9]; fleas [14]) or limited localities or habitats [4,6-9,12,15-22]. In surveys, rat-borne ectoparasites are generally categorized into four major groups, i.e. mites, fleas, ticks, and lice. Rat-borne mites, a highly diverse and specialized group, are further divided into chiggers, mesostigmatid mites, listrophorids and myobiids. Other published works related to the ectoparasite surveys include (i) descriptions of new species [23-25] and other taxonomic analyses [26,27], (ii) studies of ectoparasite morphology [27-29] and habitat [30,31], (iii) seasonal variations in diversity [17,32], and (iv) isolation of medically significant pathogens [33-35]. Other aspects such as lifecycle [36,37], and behaviour [38,39] have also been investigated but to a lesser degree.

Comprehensive surveys or reviews regarding rat-borne ectoparasites in peninsular Malaysia have seen commendable consistency in the frequency of published studies on the local rat-borne ectoparasite fauna since the early 1930’s [18,40-42] with the notable works in recent years [21,22,43,44]. Therefore, the objective of this review is to compile and correlate the available information on ectoparasite-host interactions and ectoparasite behaviour. This will lead to better perspectives on the epidemiology of related diseases as well as identify gaps in knowledge which may act to stimulate future studies.

## Review

### Mites

#### Chiggers

Following Gater’s study in 1932 [42], scientific interest in rat-borne ectoparasites was ignited by a concerted effort from the late 1940’s to early 1950’s [43-45] to investigate the epidemiology of scrub typhus in the Asia-Pacific Region. It was quickly established that chiggers (Arachnida: Trombiculidae), particularly *Leptotrombidium deliensis*, are the primary vectors for the disease. The incidences of chiggers were found to be closely associated with the presence of wild rats prompting subsequent studies to examine the ecological distribution of both chiggers and their hosts [16,37,45-47].

It soon became clear that chiggers from peninsular Malaysia are a uniquely ubiquitous and diverse group being reported in virtually every local general rat-borne ectoparasite survey [6-8]. *Walchiella oudemansi*, *Leptotrombidium deliense* and *Gahrliepia fletcheri* are the more commonly observed species [37]. Chiggers then began to generate interest independently of their association with thypus and there was a sudden increase in reports on the local, often novel, species [48-50]. A substantial portion of the reports in the Malaysian Parasite series by the Institute for Medical Research have focussed on chiggers. The overwhelming collection of data concerning the morphology, ecology, hosts, behavior, population control and medical significance of local chiggers was incorporated into an important early review paper [15,37,51]. The summary of the presence of chiggers and its hosts are as described in Table 1.

**Table 1:** **Chiggers (Acari, Prostigmata, Trombiculidae) in peninsular Malaysia**

**Species**	**Host**
*A. (A.) audyi* (Womersley, 1952)	*Lenothrix canus, L. sabanus, R.t. jalorensis*
*A. (A.) calcar* Nadchatram and Domrow, 1964	Rat
*A. (A.) ctenacarus* Domrow, 1962	*R.t.jalorensis*
*A. (A.) daria* (Traub and Audy, 1954)	Rat
*A. (A.) dentata* Nadchatram and Domrow, 1964	Rat
*A. (A.) globosa* Nadchatram and Domrow, 1964	*Tree and ground rats*
*A. (A.) indica* (Hirst, 1915)	*R.r.diardii, R.t.jalorensis*
*Cheladonta (Susa) debilis* (Gater, 1932)	*Rattus annandalei, R.t. jalorensis, L. sabanus, S. muelleri*
*Doloisia alticola* (Audy and Nadchatram, 1957)	*M. alticola*
*D. brachypus* (Audy and Nadchatram, 1957)	*M. surifer*
*D. browningi* (Audy and Nadchatram, 1957)	*B. bowersi*
*D. domrowi* (Audy and Nadchatram, 1957)	*M. surifer*
*D. harrisoni* (Audy and Nadchatram, 1957)	*B. bowersi, L. sabanus, R.t. jalorensis, M. rajah, Maxomys whiteheadi, S. muelleri*
*D. intermedia* (Audy and Nadchatram, 1957)	*M. surifer*
*D. jadini* (Audy and Nadchatram, 1957)	*B. bowersi, L. edwardsi, L. sabanus, M. rajah*
*D. owenevansi* (Audy and Nadchatram, 1957)	*M. rajah*
*D. varmai* (Audy and Nadchatram, 1957)	*B. bowersi, M. rajah, M. whiteheadi*
*Gahrliepia (G.) cetrata Gater, 1932*	*L. edwardsi, L. sabanus, S. muelleri*
*G.(G.) ciliata* Gater, 1932	Rat
*G.(G.) fletcheri* Gater, 1932	*R. argentiventer, Rattus exulans, L. sabanus, S. muelleri*
*G.(G.) insigne* Womersley, 1932	*B. bowersi, L. edwardsi, L. sabanus, M. alticola, M. rajah*
*G.(G.) neterella* Traub and Morrow, 1955	*B. bowersi, L. edwardsi, L. sabanus,*
*G.(G.) ornata* Womersley, 1952	*L. sabanus, S. muelleri*
*G.(G.) picta* Traub and Morrow, 1955	*B. bowersi, L. sabanus,*
*G.(G.) rutila* Gater, 1932	*B. bowersi, L. edwardsi, L. sabanus, R.t. jalorensis,*
*G.(G.) tessellata* Traub and Morrow, 1955	Rat
*Gahrliepia (Schoengastiella) argalea* Traub and Morrow, 1957	*L. sabanus*
*G.(S.) arona* Traub and Audy, 1954	*M. whiteheadi, S. muelleri*
*G. (Walchia) alpestris* Traub and Evans, 1957	*B. bowersi*
*G. (W.) brennani* Womersley, 1952	Rat
*G. (W.) cupa* Traub and Evans, 1957	*M. surifer*
*G. (W.) cuspidata* Traub and Evans, 1957	*L. sabanus*
*G. (W.) disparangus* (Oudemans, 1910)	*M. whiteheadi*
*G. (W.) enode* (Gater, 1932)	*S. muelleri*
*G. (W.) ewingi* (Fuller, 1949)	*M. whiteheadi*
*G. (W.) lewthwaitei* Gater, 1932	*M. rajah, M. whiteheadi, R. argentiventer, R. exulans, R.t. jalorensis*
*G. (W.) pingue* (Gater, 1932)	*L. sabanus, L. edwardsi, M. rajah, M. whiteheadi, Niviventer rapit*
*G. (W.) rustica* Gater, 1932	*L. sabanus, M. surifer*
*G. (W.) simulata* Traub and Evans, 1957	*B. bowersi, L. edwardsi, M. alticola*
*G. (W.) turmalis* Gater, 1932	*B. bowersi, L. edwardsi, L. sabanus*
*G. (W.) ventralis* Womersley, 1952	*B. bowersi*
*Heaslipia gateri* (Womersley and Heaslip, 1943)	Rat
*Helenicula mutabilis* (Gater, 1932)	*L. sabanus, S. muelleri*
*Kayella novita* (Audy and Womersley, 1957)	*M. rajah*
*Schoutedenichia bisetosa* Domrow, 1962	*Rat*
*S. vercammeni* Audy, 1956	*L. sabanus*

#### Mesostigmatid and other mites

Among the rat-borne ectoparasites groups; rat mites (Arachnida) have received the most attention. Rat mites are a highly diverse and specialized group that includes chiggers (see above), mesostigmatids, listrophorids and myobiids. Major general works include Domrow and Nadchatram [10], Domrow [25] and Ismail *et al*. [11]. Lately, attention has begun to shift from chiggers toward the mesostigmatid mites [11,13], another similarly widespread and species-rich group. These recent studies have accumulated information on host distribution, diversity and morphological characteristics. *Laelaps echidninus*, *Laelaps nutalli*, *Laelaps sedlaceki L. turkestanicus L. sanguisugus* and *Longolaelaps whartoni* are some of the more common rat-borne mesostigmatids in peninsular Malaysia [21,44]. Other less common species include *Haemolaelaps gallinarii* [52]; *Haemolealaps argentiventer* [53]; *Haemogamasus liberiensus* and *Tricholealaps vitzthumi* [54,55].

In contrast, information on the distribution and prevelance of the other two rat mite groups, the listrophorids (Table 2) and myobiids (Table 3) is still preliminary with only eighteen and ten species having been recorded respectively. This is possibly due to the fact that these ectoparasites have shown little or no medical and ecological significance. Listrophorids infestations among the local wild rats have only been reported occasionally [4,5]. Even less has been reported about diversity and distribution of rat myobiids [4]. Presently, two publications by Fain and collagues [56,57] remain the definitive references on the local rat-borne listrophorids and myobiids.

**Table 2:** ***Listrophoroides*** **Hirst, 1923 (Acari, Astigmata, Atopomelidae) of rats in peninsular Malaysia**

**Species**	**Host**	**Locality**
*Listrophoroides (Listrophoroides) biexcavatus* (Fain, 1979)	*Maxomys rajah*	Besut, Terengganu Mentakab, Pahang
*L. (L.) borneoensis* (Fain, 1970)	*Sundamys muellari*	Ulu Langat, Selangor
	*Rattus argentiventer*	Ulu Jenderam, Selangor
*L. (L.) brachypyx* (Fain, 1974)	*Rattus tiomanicus*	Bukit Lanjan, Selangor
*L. (L.) cocoensis* (Fain, 1976)	*Maxomys rajah*	Besut, Terengganu, Mentakab, Pahang.
*L. (L.) eudrilus* (Fain, 1976)	*Maxomys surifer*	Besut, Terengganu Perak
*L. (L.) hemistriatus* (Fain, 1976)	*Maxomys surifer*	Ulu Setia, Terengganu
*L. (L.) hongkongensis cremoriventer* (Fain, 1976)	*Niviventer cremoriventer*	Bukit Besar, Besut, Terengganu
*L. (L.) maculatissimus* (Fain, 1979)	*Maxomys rajah*	Besut, Terengganu, Terengganu; Mentakab, Pahang
*L. (L.) neobifidus* (Fain, 1979)	*Maxomys rajah*	Bukit Lanjan, Selangor
	*Maxomys surifer*	Ulu Setia, Terengganu
*L. (L.) pahangi* (Fain, 1974)	*Leopoldmys edwardsi*	Ulu Langat, Selangor; Mt Brinchang, Cameron Highland, Pahang
	*Leopoldmys sabanus*	Gunong Benom, Pahang; Terengganu
*L. (L.) ptilocereus* (Fain, 1970)	*Sundamys muellari*	Ulu Langat, Selangor
*L. (L.) rajah* (Fain, 1974)	*Maxomys rajah*	Ulu Setia, Terengganu.
*L. (L.) taxophallus* (Fain, 1976)	*Maxomys surifer*	Ulu Setia, Terengganu
*L. (L.) uluensis* (Fain, 1979)	*Maxomys surifer*	Ulu Setia, Terengganu
*L. (Marquesania) cucullatus *(Trouessart 1893)	*Rattus argentiventer*	Ulu Jenderam, Selangor
	*Rattus exulans*	Ulu Jenderam, Selangor
	*Rattus t. jalorensis*	Bukit Lanjan, Selangor
*L. (M.) lativentris* (Fain, 1981)	*Sundamys muellari*	Terengganu; Gombak
		Forest Reserve, Selangor
		Bukit Lanjan, Selangor
*L. (M.) sculpturatus* (Fain, Nadchatram and Lukoschus 1981)	*Sundamys muellari*	Selangor

**Table 3:** **Myobiidae (Prostigmata) from rats in peninsular Malaysia**

**Species**	**Host**	**Locality**
*Radfordia (Radfordia) ensifera* (Poppe, 1896)	*Rattus rattus diardii*	Gombak Forest Reserve, Selangor
	*Rattus tiomanicus jalorensis*	Bukit Lanjan Forest Reserve, Selangor; Subang Forest Reserve, Selangor
*R. (R.) ensifera jalorensis* (Fain, Lukoschus and Nadchatram, 1980)	*Rattus tiomanicus jalorensis*	Bukit Lanjan Forest Reserve, Selangor
*R. (R.) hornerae* (Domrow, 1963)	*Leopoldmys sabanus*	Templar’s Park, Selangor
*R. (Rattimyobia) perakensis* (Fain, 1973)	*Maxomys rajah*	Bukit Lanjan Forest Reserve, Selangor; Kampong Awak, Temerloh, Pahang
	*Maxomys surifer*	Perak
*R. (Rattimyobia) acinaciseta* (Wilson, 1967)	*Maxomys rajah*	Bukit Lanjan Forest Reserve, Selangor; Mentakab, Pahang
*R. (Rattimyobia) pahangensis* (Fain, Lukoschus and Nadchatram, 1980)	*Maxomys inas*	Mt. Brinchang, Cameron Highlands, Pahang
	*Niniventer bukit*	
*R. (Rattimyobia) selangorensis* (Fain, Lukoschus and Nadchatram, 1980)	*Maxomys whiteheadi*	Subang Forest Reserve, Selangor
*R. (Rattimyobia) subangensis* (Fain, Lukoschus and Nadchatram, 1980)	*Maxomys rajah*	Subang Forest Reserve, Selangor
*R. (Graphiurobia) chiropodomys* (Fain, 1974)	*Chiropodomys gliroides*	Gombak Forest Reserve, Selangor
*Myobia (Myobia) malaysiensis* (Fain, Luoschus and Nadchatram, 1980)	*Chiropodomys gliroides*	Gombak Forest Reserve, Selangor

### Fleas

Documentation of rat-borne fleas (Insecta: Siphonaptera) and ticks (Arachnida: Ixodida) also began during the 1940s and 1950s [58], and continued intermittently over the following years [13,18,21,41]. There has only been two long-term surveillance studies on the incidence of fleas among local small mammals [14,59]. Both studies recognized *Xenopsylla cheopis* as the most prevalent rat-borne flea in the region. Zahedi *et al*. [60] showed that urban rat populations had a higher flea index compared to semi-urban rat populations. They also reported 6% of the rats trapped in the city harboured the cat flea, *Ctenocephalides felis* [60]. Other species include *Stivalius ahalae* [6], *Rothschildiana smiti* [23] and interestingly, *Xenopsyllaastia*, a common cosmopolitan species, has been observed only once [3]. *Stivalius jacobsoni* on *Niniventer rapit*, *Stivalius klossi* on *Niniventer cremoriventer* and *Paraceras* sp. on *Maxomys edwardsi* have been reported by Traub [58].

### Ticks

The first major study on rat-borne ticks from peninsular Malaysia was conducted by Kohls [9], who compiled a host-parasite checklist and a comprehensive taxonomic key. The study provided the basis for further investigations into the host distribution of rat-borne ticks in the region [61,62]. Extensive collections have revealed wild terrestrial rats to be a common, perhaps preferred, host for immature ticks (e.g. *Amblyomma* spp., *Haemphysalis* spp. and *Dermacentor* spp.), while *Ixodes granulatus* was the only adult tick found on rats [9-12,16,17,63]. Interestingly, Nadchatram *et al*., 1966 recorded *Amblyomma helvolum*a species usually found on reptiles on a rat, *Leopoldamys sabanus* [16]. However, difficulties identifying immature rat-borne ticks beyond the genus level have hindered efforts to associate tick infestations with the behavioral and ecological data on their hosts.

### Lice

Studies concerning rat-borne lice have focused on morphology for taxonomic identification and phylogenetics [13,28,64]. Presently, *Hoplopleura pacifica, H. dissicula, H. pectinata* and *Polyplax spinulosa* have been recorded from peninsular Malaysia [21,65]. Lice have only been included occasionally in the general rat-borne ectoparasites surveys [6,7,18,21,22,41].

### Breaking the taxonomic boundaries

Early rat-borne ectoparasite surveys showed a propensity to limit their focus on specific groups, as evident in the review by Audy [15]. As a result, the overall rat-borne ectoparasite community has been largely neglected and the intercommunity population dynamics are poorly understood. Recently there have been attempts to redress the situation [4,11,41].

Perhaps for pragmatic reasons, past researchers seemed content in focusing on cataloguing the ectoparasites’ host distribution and describing novel species. The majority of reports are strictly short-term field observations and prevalence studies, and though several extensive, noteworthy works were produced [9,28,48], the seemingly perfunctory approach has restricted further insights into the factors influencing the development, behaviour and transmission of rat-borne ectoparasite species. Additionally, besides for chiggers and ticks, there had been virtually no experimental investigations conducted on local rat-borne ectoparasites [36,38,66]. This may be due to the fact rat-borne ectoparasites do not represent immediate zoonotic risks.

A study by Ho and Krishnasamy [12] warrants a special mention as a survey in which both the ectoparasite and endoparasite of small mammals from Taman Negara was recorded concurrently. Although the study was little more than a host-parasite list, it raises the issue of the probable links between concurrent rat-ecto and endoparasite infections, in which the ectoparasites may serve as intermediate hosts for certain helminthes.

### Identification and morphology

Identification of rat-borne ectoparasites had thus far been entirely based on the morphological characteristics of the specimens using available keys and published reference manuals [9,28,67]. Morphological studies therefore placed an emphasis on in-depth and reliable descriptions of the local species and are often accompanied with detailed graphics [25,28]. Perhaps curiously, the most detailed morphological descriptions of the rat-borne ectoparasite fauna of peninsular Malaysia were those of rat-borne lice and ticks while the morphology of rat myobiids and listrophorids from peninsular Malaysia have yet to be systematically and thoroughly described.

### Distribution and prevalence patterns

Rat-borne ectoparasites have been reported from various habitats throughout peninsular Malaysia, including primary lowland forest [11,19], secondary forest [5], montane forests [29,68], cities [14,18], cultivated fields [69,70] and coastlines [31]. The majority of collections have been from primary rainforests but several studies have also conducted concurrent investigations of more than one habitat [10,28,40].

These surveys have enabled comparions of the diversity of rat-borne ectoparasites from different habitats across peninsular Malaysia and revealed that generally, each ectoparasite species is restricted to a certain habitat [71-73]. For example, rat-borne fleas and lice were commonly recorded in urban areas but were rarely recorded anywhere else [60]. The rat-borne ectoparasite distribution is therefore assumed to be closely linked to the host species’ habitat specialisation. Audy [27] remarked that for chiggers, “habitat-specificity may give an appearance of host-specificity, and occasionally it may not be possible to explain findings confidently by one explanation or the other”. Similar thoughts were also expressed by several authors [32,44,66,74] and while it may seem reasonable to assume that a degree of host specificity exists, its actual role, as well as the extent of its effects, still needs to be clarified.

The impact of human activities on the disease patterns of rat-borne infections has only been briefly remarked upon [66] and anthropogenic changes to the habitat of rats appear to have unpredictable consequences for the prevalence and distribution of rat-borne ectoparasites [66,70,75,76]. The severity and long-term effects are still largely unclear. Surveys in Taman Negara [12,19,35] and Gombak Forest Reserve [4,11] could provide the basis for long-term studies that would enable future researchers to detect changes in the diversity and intensity of rat ectoparasite infestations that may occur as a result of human impact. Traub and Wisseman [70] hypothesized that rats occupying permanent nests would exhibit higher mite burdens because mites, particularly mesostigmatids, are nest dwellers and that infestations are acquired during the hosts’ time in the nest. The authors further speculated that spiny- furred rats provide a more conducive environment for mites, in contrast to soft-furred and semi-spiny-furred hosts, thereby contributing to the supposed higher mites loads in such hosts. However, no studies have set out to futher test this hypothesis.

There have been no attempts to investigate the effects of the age and sex of hosts on ectoparasite burdens and transmission, but studies have indicated a link between host size and parasite abundance [32].

The effect of seasonal changes and rainfall on ectoparasite burdens have only been briefly explored. It has been hypothesized that dry conditions may have an adverse effect on chigger infestations [32,51]. Lim [17] was unable to conclusively demonstrate the association of rainfall with immature tick infestations, while Singh *et al*. [14] likewise did not observe any relationship between rainfall and flea indices. The altitudinal distribution of rat-borne ectoparasites has been investigated by Hoogstraal *et al*. [77] and Nadchatram [68], who found ticks were found in reasonable numbers at the higher altitudes, while chiggers were virtually absent. The actual effects of altitude on the distribution of rat-borne ectoparasites are however not readily apparent and warrant further study.

### Vectors of human diseases

Rat ectoparasites have long been known to be capable of transmitting human diseases such as (i) scrub typhus, (ii) murine typhus, (iii) other types of typhus, (iv) the plague, (v) *Bartonella* and (vi) various viruses. In fact, the potential threat to public health has been the major justification for many studies [35].

(i) Scrub typhus, a rickettsial disease transmitted through the bite of an infected chigger, is arguably one of the more well-known diseases associated with the rat-borne ectoparasites in peninsular Malaysia. It is a common cause of fever in rural populations [78,79]. The distribution of scrub typhus has been extensively studied, and is generally associated with cultivated fields and secondary forests, and its epidemiology is linked to human behaviour and occupation [80]. *Leptotrombidium deliensis*, frequently found infesting wild rats living within or near the vicinity of agriculatural land and secondary forests, is the major vector for *Orientia tsutsugamushi*, the causative agent for the disease [37,58]. Other chigger species have also been implicated in maintaining and transmitting the disease [35,81,82]. An exhaustive and highly comprehensive review of scrub typhus and various related factors by Traub and Wisseman [51] concluded that chiggers of the subgenus *Leptotrombidium* associated with rats from the genus *Rattus* were able to penetrate new areas and habitats and diversify, and as the chiggers adapted to other hosts in the new regions, such mammals became secondarily involved in the ecology of the rickettsiosis.
(ii) Murine typhus, also known as urban typhus, is another rat-borne disease from the typhus group. The causative agent is *Rickettsia typhi* and infection can be transmitted from rats to humans though rat-borne fleas, *Xenopsylla cheopis* [83]. Murine typhus is still considered uncommon despite indications that the disease is more widespread than previously thought [34,80,84].
(iii) There has been indications of a “jungle cycle” scrub typhus in peninsular Malaysia [51,58,85]. Cadigan Jr *et al*. [86] and Robinson *et al*. [87] linked the length of residence in forested areas with the infection rate among humans. Though the existence of other scrub typhus strains remains in question, the risks of typhus from primary forests is estimated to be considerably less compared to areas typically associated with the disease [35]. Occurrences of tick typhus or spotted fever group rickettsiae (SFGR), another rickettsioses associated with rat-borne ectoparasites, have also been recorded in peninsular Malaysia [33,80,84]. Marchette [33] implicated *Ixodes granulatus* and *Dermacentor auratus* in their transmission.
(iv) Rat-borne fleas are also known vectors of *Yersinia pestis*, the bacterial agent of plague. While there has been no outbreaks in recent years in peninsular Malaysia, plague is endemic in other Southeast Asian countries, including Indonesia [88,89], Thailand [90], Vietnam [91] and Myanmar [92]. The first case of plague in Malaysia occurred in Penang in 1896 and the most recent case was in Perak in 1928 [3].
(v) The occasional rat-borne flea (*Ctenocephalides felis*) and rat-borne tick (*Ixodes granulatus*) are also often implicated in the natural maintenance of various species of *Bartonella*. A recent study observed five *Bartonella* species (*Bartonella tribocorum, B. rattimassiliensis, B. coopersplainsensis B. elizabethae* and *B. queenslandensis*) circulating in the rat population in Kuala Lumpur [93].
(vi) Rat-bourne ticks are also known hosts of the Langat Virus Langat Virus [94], the forest cycle of Q-fever [33], and the Lanjan Virus [95]. Lanjan virus has been isolated from immature *Dermacentor* [95,96].

Aside from harbouring and transmitting dangerous pathogens, the ectoparasites themselves are also capable of causing skin irritations for humans. Among the local rat-borne ectoparasites, mesostigmatids i.e. *Ornithonyssus bacoti*, *Laelaps echidninus* and *Laelaps nuttalli*, have been reported as attacking humans, resulting in varying degrees of dermatitis [67,97]. Chiggers are also known to occasionally cause irritations commonly known as scrub itch [12].

Human health concerns associated with chiggers are rural in nature, confined to secondary forest and plantations i.e. areas with low population density. Although the threat of chiggers on human health is fortunately limited, its impact on ecotourism and other daily human activities are often overlooked. Rat-borne fleas appear to pose the highest health risk to humans as their rat hosts and distribution are concentrated within or near high-density human habitations but there has been no serious outbreaks of diseases associated with rat-borne fleas. Likewise the medical significance of rat-borne ticks on public health is presently strictly academic. Despite being shown to be capable of harbouring several dangerous pathogens, there has yet to be any verified reports of tick related infections in peninsular Malaysia. The other ectoparasites groups, the rat-borne lice, myobiids and listrophorids also are presently of no known medical importance [98]. Further investigations should be initiated to update and verify their significance to public health.

To summarize, the collective impact of the local rat-borne ectoparasites on public health is presently low, perhaps even negligible. However, the above studies have shown that these ectoparasites harbour a number of serious pathogens, and could potentially become major health risks in the future. To circumvent this, population dynamics of rat-borne ectoparasites and the epidemiology need to be better understood.

### Major gaps in present knowledge of rat-borne cctoparasites of peninsular Malaysia

#### Quantitative data and statistical analyses

Despite consistent attempts to survey and catalogue the various rat-borne ectoparasite species throughout the region, the collection of quantitative data and statistical analysis of the infestations have been surprisingly inadequate. Though a few surveys did calculate the prevalence of the infestations [4,5,18] and mean intensity values [17,32], measures of variability seem mostly to have been disregarded. With the exception of the work of Ismail *et al*. [11], diversity and aggregation indices, commonly found in the more recent international parasitological publication, have failed to be incorporated into the peninsular Malaysia investigations. Most surveys conducted have been little more than a host-parasite checklists.

1. Interspecific and intraspecific interactions
  Inter- and intraspecific interactions of different ectoparasite species have never been fully explored. As it is commonly observed that wild terrestrial rats often harbour concurrent ectoparasites infections it would therefore be of great interest to observe how these ectoparasites interact and affect one another within the limited space (e.g. crowding effects, competition, etc.).
2. Natural resistant of hosts
  While briefly mentioned in Boese’s study [99], the pathological aspects of ectoparasite infestation of rats are poorly documented. Virtually no information is available on natural resistance and immunological reactions of the host with ectoparasite infestations.
3. Niche specialization
  There have been observations that certain chiggers species from peninsular Malaysia occupy a specific site within their hosts, such as the nasal cavities [100]. These unique microhabitats have since become an important characteristic for species identification [26,101]. The existence of site specificity is intriguing and reflects the degree of interspecific interactions that may occur in concurrent infestations. Surprisingly however, the phenomenon was not investigated further and it is not known if the species of other ectoparasite groups exhibit a similar behaviour.
4. Morphology and identification
  Most studies carried out by scientists in peninsular Malaysia adopt the conventional method for ectoparasite identification i.e using morphological keys. Using this method only experienced taxonomists are able to distinguish species reliably. The availability of molecular biology as a tool to assist identification has become common place [102], however, there is neither interest nor urgency among researchers to conduct molecular characterization on the local rat-borne ectoparasite species.

## Conclusions

Considerable attention has been given towards the ectoparasites of the wild, terrestrial rats in peninsular Malaysia. The majority of rat-borne ectoparasitological studies were field surveys and collections of specimens from across the region although other aspects such as morphology, host distribution and medical significance have also been investigated. Local rat-borne ectoparasite studies often focus on selected groups, particularly chiggers due to their impact on human health, although in recent years attention has slowly shifted towards the overall ectoparasite diversity. Studies on rat-borne ectoparasites have been invaluable in the fields of ecology and public health.

Despite the number of studies that have been published, a number of related issues have to be addressed. Distribution patterns of certain rat-borne ectoparasites still remains unknown. Factors influencing infestations as well as changes to the population distribution over time are also poorly understood. Additionally, the quantitative aspects of ectoparasite infestations have also been largely neglected. It is therefore vital for researchers in the field to redevelop and reprioritize their focus towards these lesser known aspects.
